# Supplementary figures and images for: Mutualism provides a basis for biodiversity in eco-evolutionary community assembly
Source: PLoS Comput Biol. 2025 Sep 2;21(9):e1013402. doi: 10.1371/journal.pcbi.1013402 (PMC12416848; doi:10.1371/journal.pcbi.1013402)

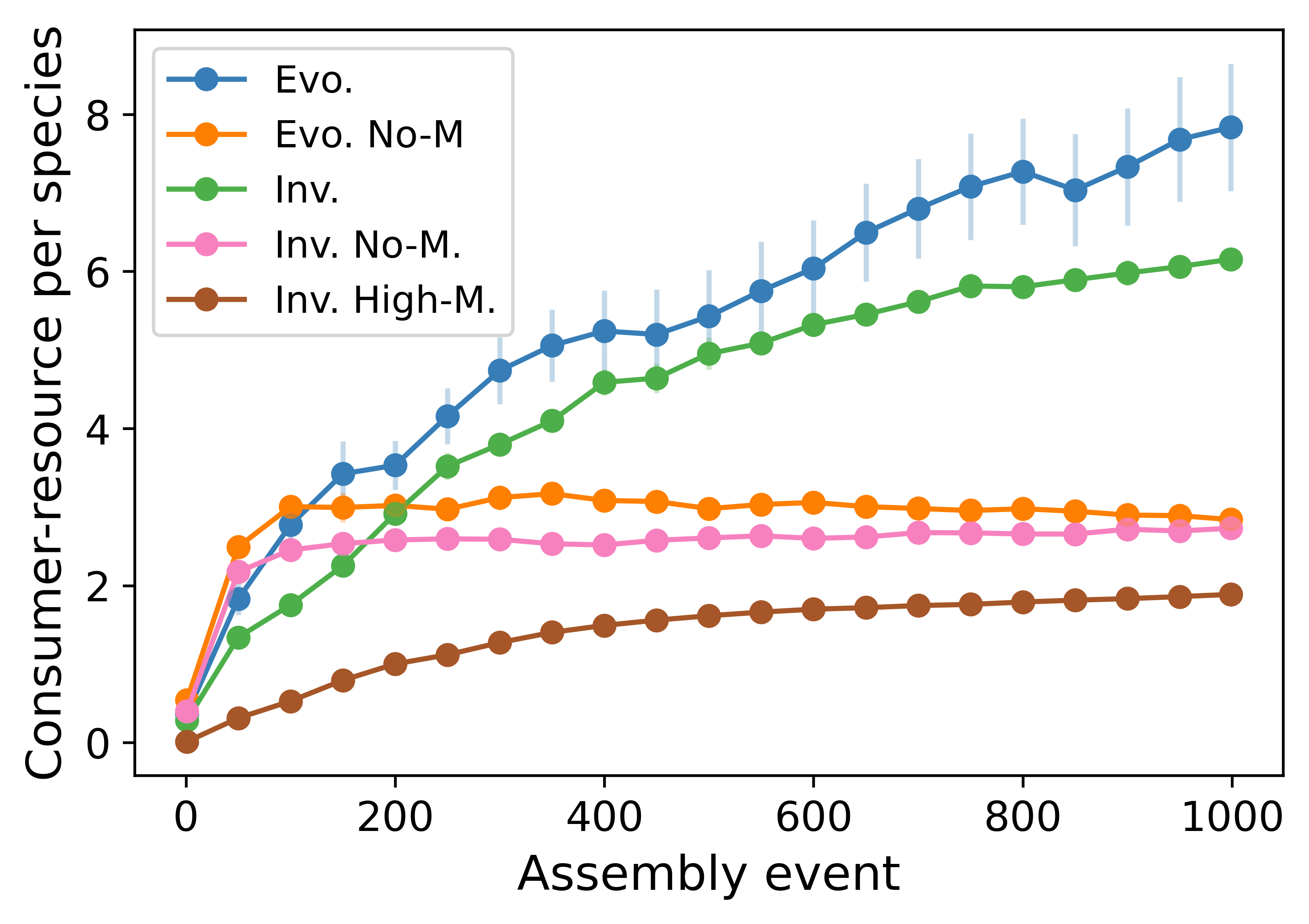

Supplement: S1 Fig — During each assembly event, a new successful (i.e. capable of growing and establishing) species is added to the community. Evo. scenarios are assembled via speciation, and Inv. scenarios are assembled via invasion. No-M scenarios do not feature mutualisms, while in Inv. High-M invading species have a proportion of mutualisms uniformly chosen between 0.8 and 1. The plots show the average values of 15 samples for every 50 assembly events (dots) and the vertical lines show the standard errors, calculated as the standard deviation divided by the square root of the number of samples. Each sample is a simulation of the entire assembly process for a scenario. The average number of consumer-resource interactions sustained by each species is higher when high proportions of mutualism are selected than when mutualism is not present. Evolution (i.e. speciation) enhances this effect, in comparison with invasion. Parameter values: interaction strengths were drawn from a half-normal distribution of zero mean and a standard deviation 0.2, and strength for consumers was made no larger than the strength for resources. Communities started with 5 non-interacting species. New species were given initial abundances equal to the extinction threshold xext=10−6. Connectance of invading species was drawn from a uniform distribution between 0.05 and 0.5. In speciation, offspring species had up to 5 interactions differing from the parent species, chosen randomly (Δ=5, see Methods). Handling times for mutualism and consumer-resource was 0.1. (PNG) [file pcbi.1013402.s001.png]

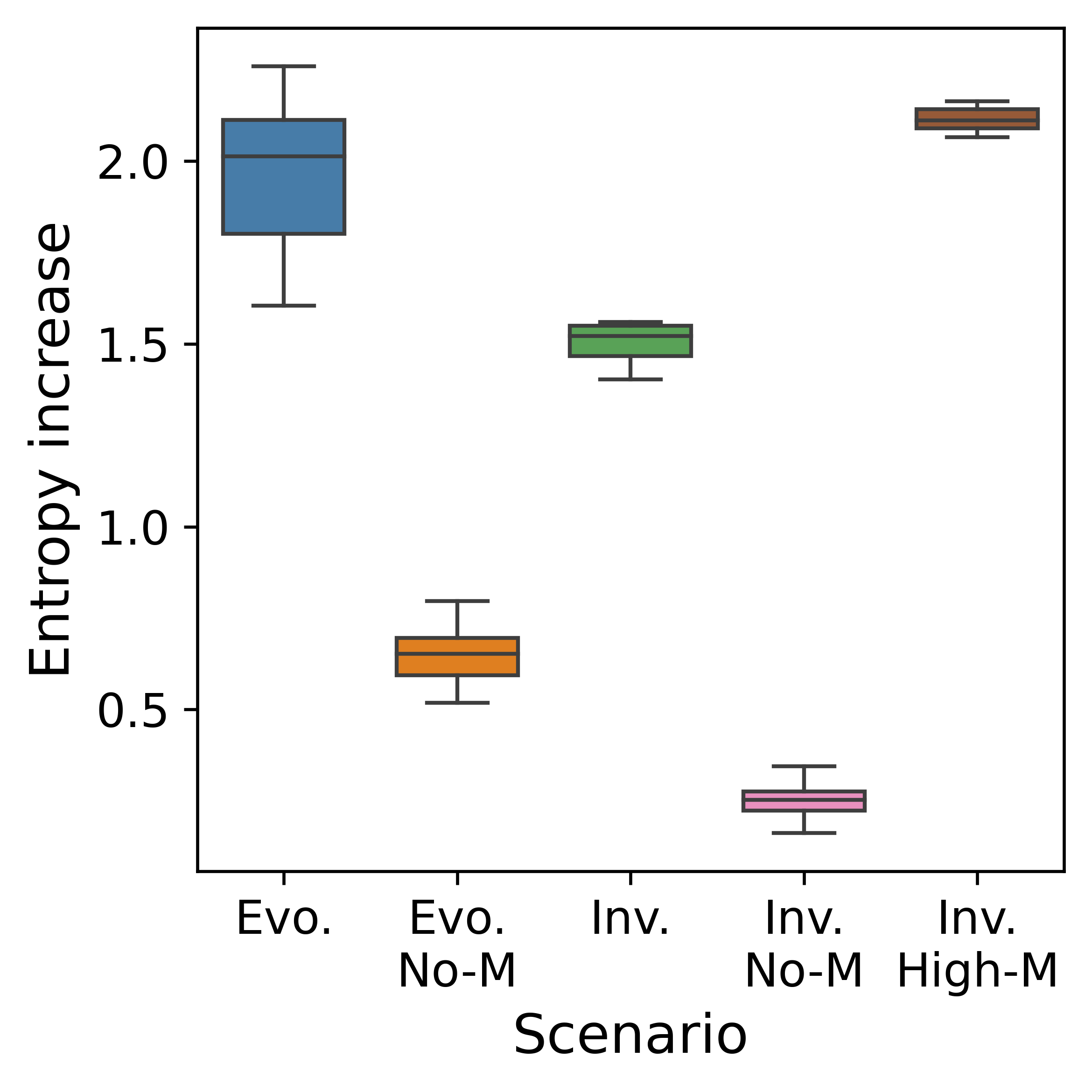

Supplement: S2 Fig — Evo. scenarios are assembled via speciation, and Inv. scenarios are assembled via invasion. No-M scenarios do not feature mutualisms, while in Inv. High-M invading species have a proportion of mutualisms uniformly chosen between 0.8 and 1. Degree entropy of communities normalised by the average random network with the same richness and connectance. The random average was calculated using 50 random network samples. To calculate the entropy increase, the random average was subtracted from the degree entropy of all 15 community samples. Each sample is a simulation of the entire assembly process for a scenario. A value of zero increase corresponds to the average of the random counterparts, while positive values correspond to an increase from the value expected by chance. The degree entropy increases for all models, but a higher increase is driven by high proportions of mutualistic interactions. Mutualisms promote a higher homogeneity of degrees. (PNG) [file pcbi.1013402.s002.png]

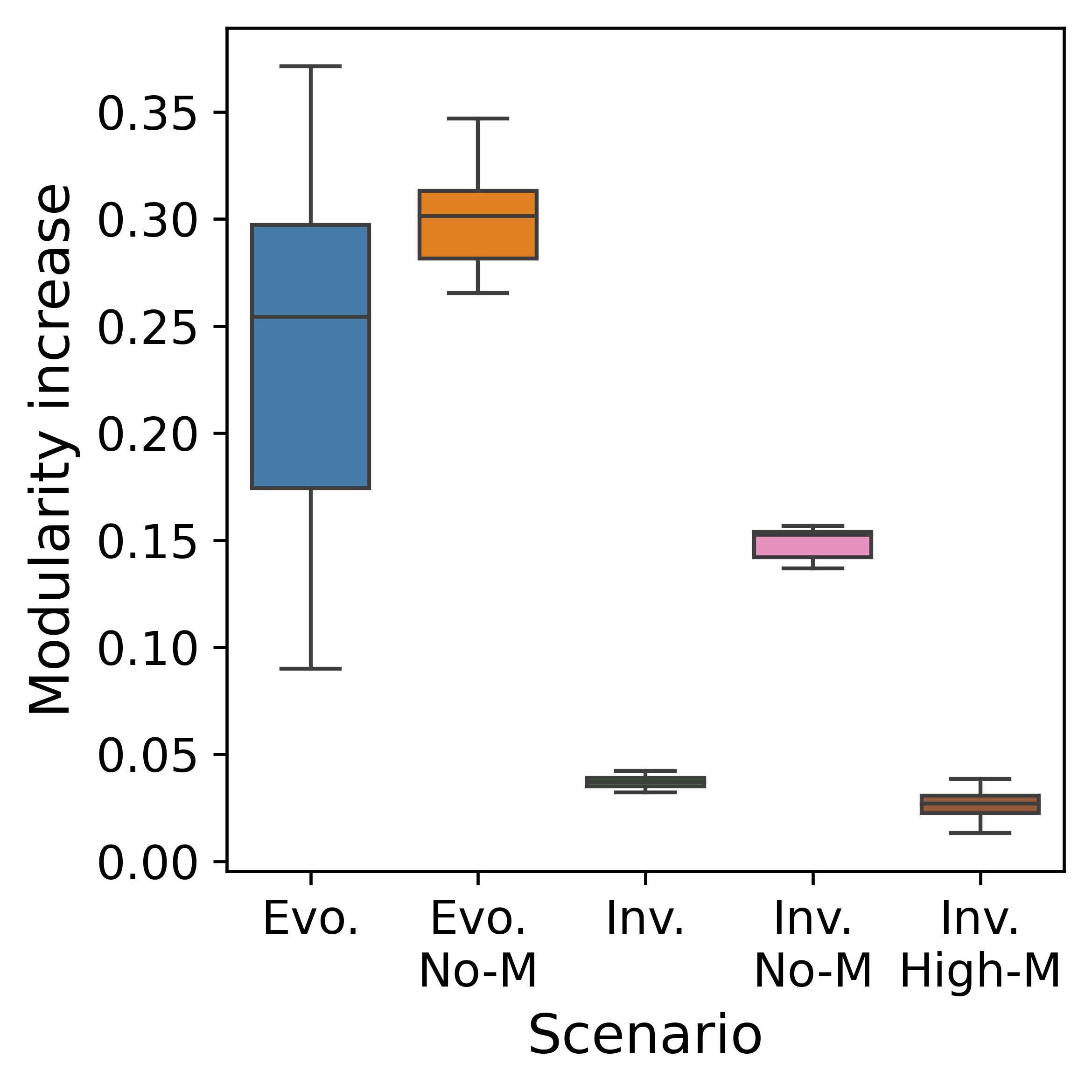

Supplement: S3 Fig — Evo. scenarios are assembled via speciation, and Inv. scenarios are assembled via invasion. No-M scenarios do not feature mutualisms, while in Inv. High-M invading species have a proportion of mutualisms uniformly chosen between 0.8 and 1. Modularity of communities normalised by the average random network with the same richness and connectance. The random average was calculated using 50 random network samples. To calculate the entropy increase, the random average was subtracted from the degree entropy of all 15 community samples. Each sample is a simulation of the entire assembly process for a scenario. A value of zero increase corresponds to the average of the random counterparts, while positive values correspond to an increase from the value expected by chance. Modularity is mainly driven by speciation, with the lack of mutualism also being responsible for an increase. Invasion models with mutualism barely increase modularity, while evolution promotes the highest increase. (PNG) [file pcbi.1013402.s003.png]

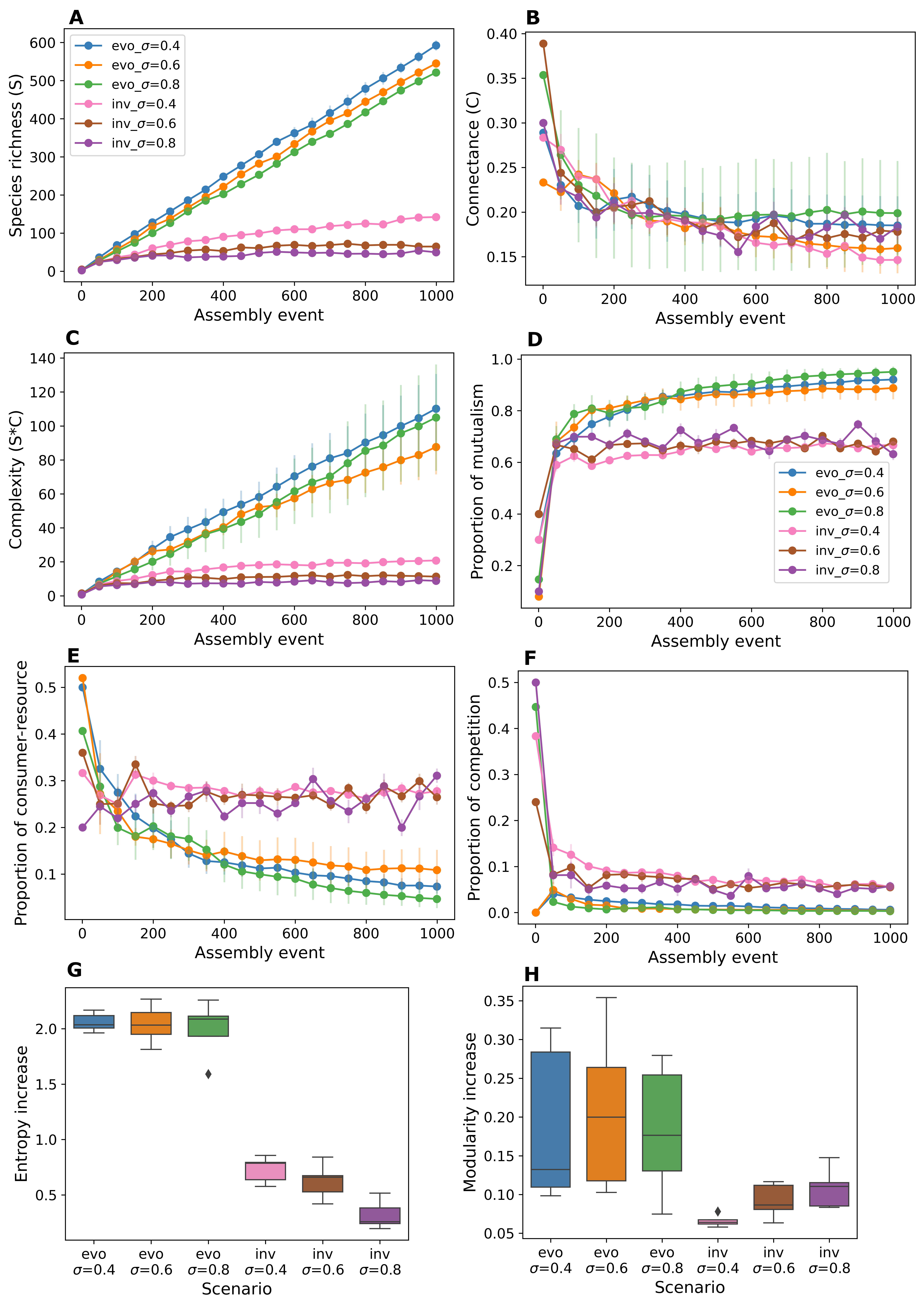

Supplement: S4 Fig — Reproduction of main results from Figs 1, 2, S2 Fig and S3 Fig for Evo. and Inv. scenarios with σ=(0.4,0.6,0.8), 5 samples each. As σ gets larger, interactions become stronger. (A-H) All results remain similar for stronger interactions. Two notable differences are that 1. variability in connectance for evolution increases for stronger interactions and 2. entropy increase becomes smaller and modularity increase becomes larger for invasion. All parameter values are the same as in the main results. (PNG) [file pcbi.1013402.s004.png]

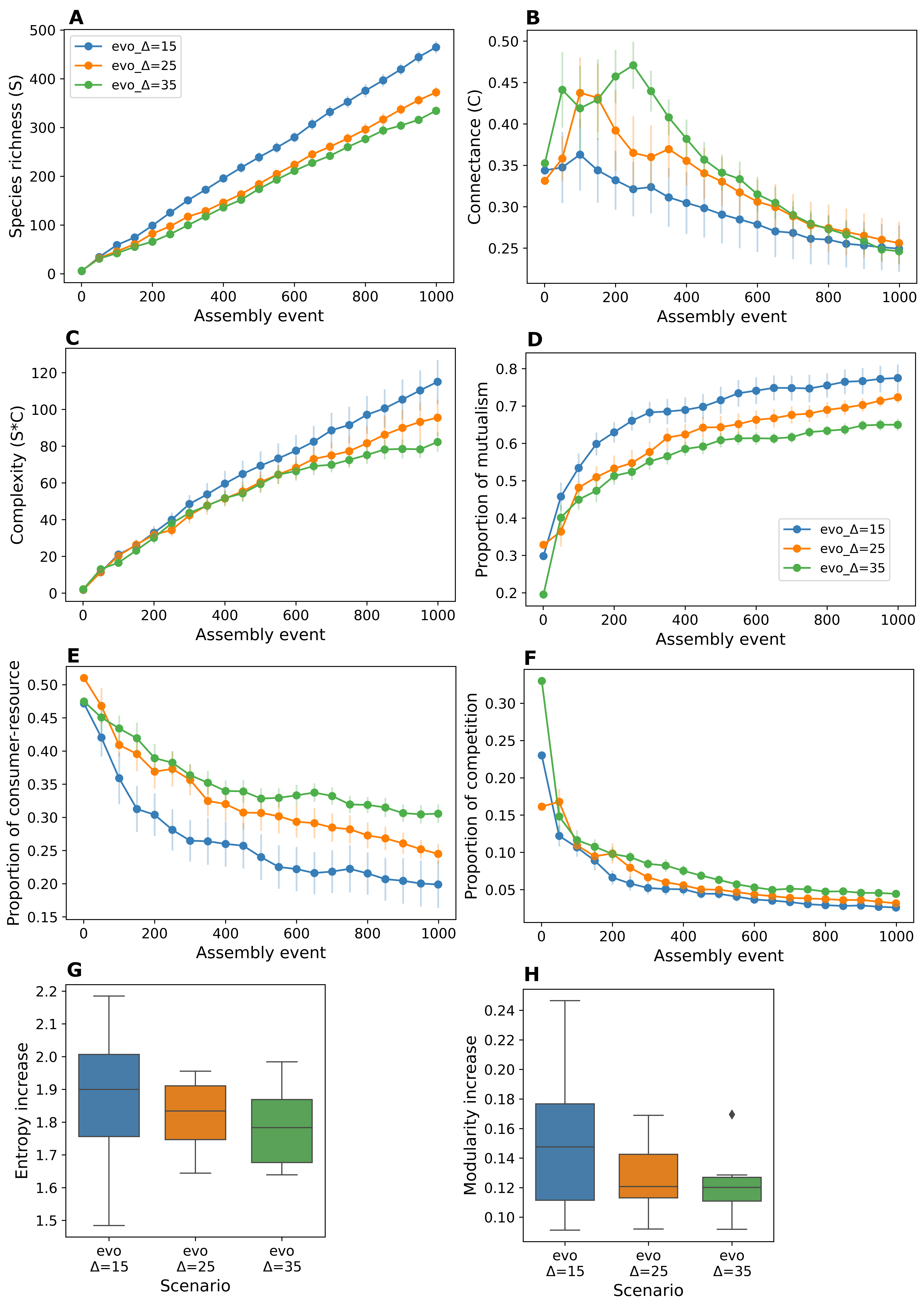

Supplement: S5 Fig — Reproduction of main results from Figs 1, 2, S2 Fig and S3 Fig for the Evo. scenario with Δ=(15,25,35), 10 samples each. As Δ gets larger and the degree of inheritance gets lower, speciation results approach the ones obtained with assembly by invasion. However, a small inheritance still generates visible differences. (A-C) Richness gets lower, resulting in less complexity, but connectance gets higher at the beginning then remains at the same level in the end. (D-H) Interaction composition and network metrics change as predicted. All parameter values are the same as in the main results. (PNG) [file pcbi.1013402.s005.png]

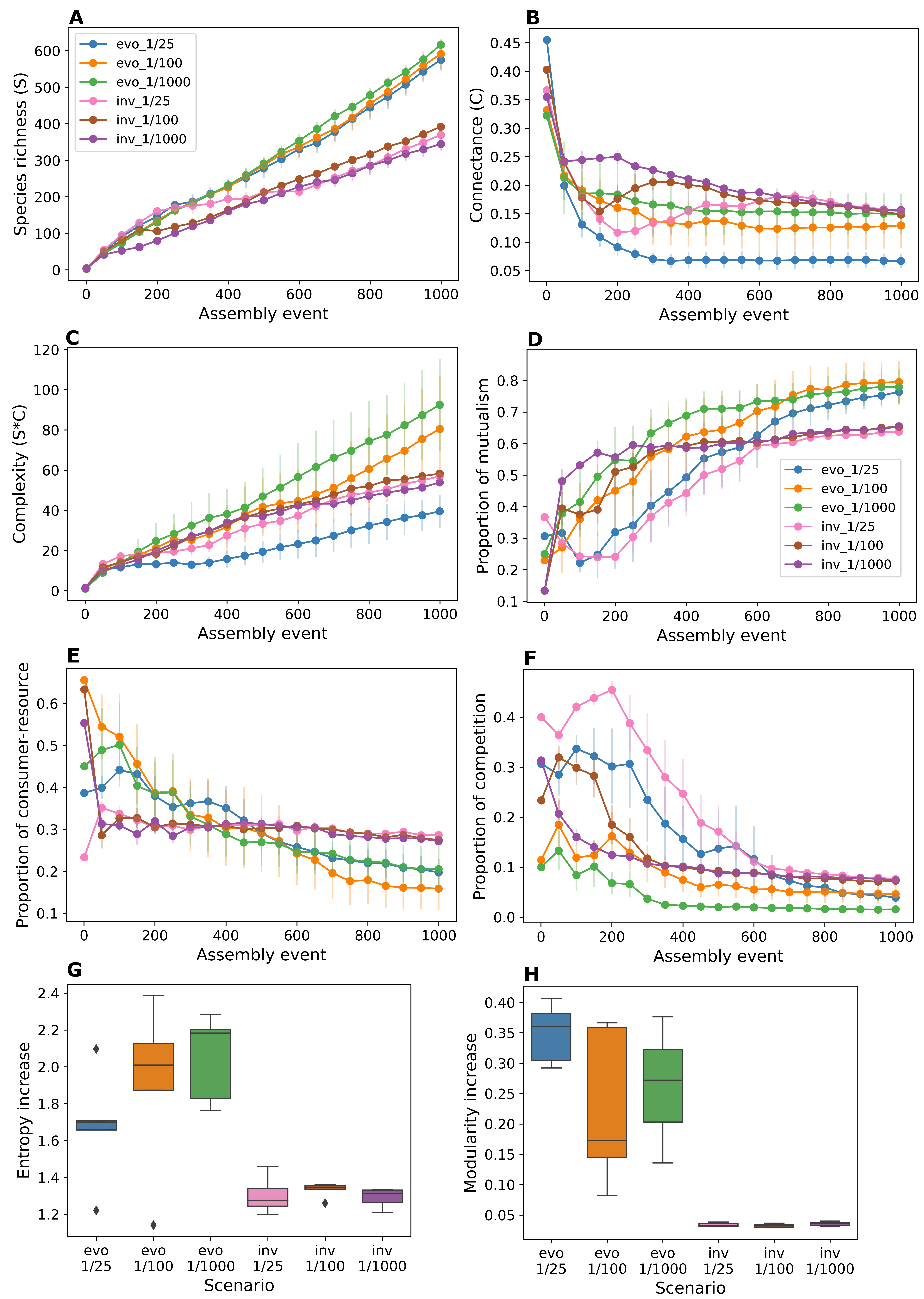

Supplement: S6 Fig — Evo. Reproduction of main results from Figs 1, 2, S2 Fig and S3 Fig for Evo. and Inv. scenarios with assembly events occurring before ecological equilibrium is attained, 5 samples each. The fastest assembly scenario is for 1 event at every 25 time-steps (1/25), then 1 at every 100 time-steps (1/100), and the slowest with 1 at every 1000 (1/1000), all with evolution or invasion happening in the same time-scale as the ecological dynamics. (A-H) All results remain the same as the ones obtained with separation of time-scales, apart from very fast evolution (Evo. 1/25). Composition of interaction types remains similar, but complexity decreases considerably as a result of a much lower connectance. All parameter values are the same as in the main results. (PNG) [file pcbi.1013402.s006.png]

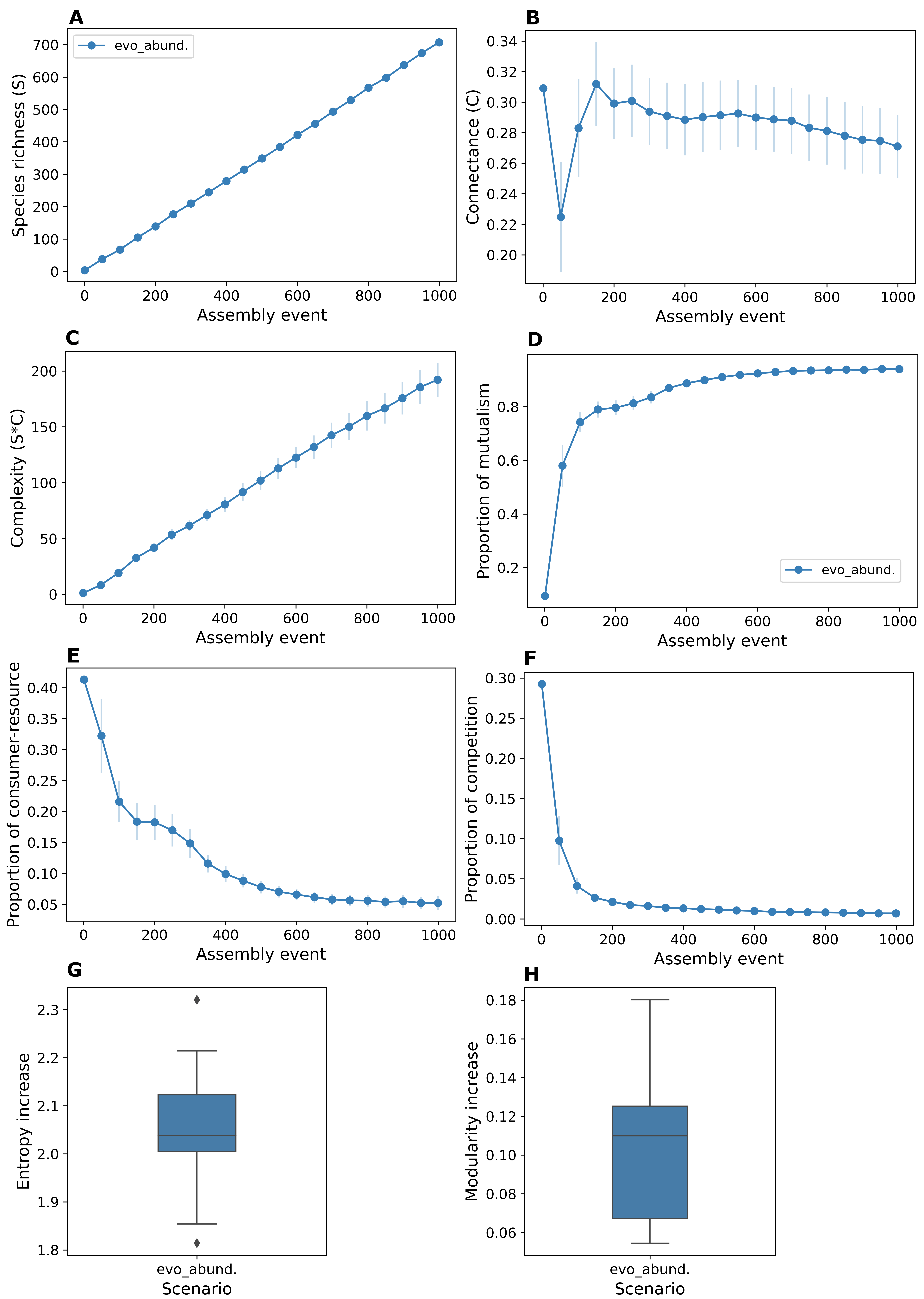

Supplement: S7 Fig — Evo. Reproduction of main results from Figs 1, 2, S2 Fig and S3 Fig for an Evo. scenario in which the probability of a species being selected as a parent species for speciation is weighted by its abundance, instead of being the same for all species, 10 samples. (A-H) All results remain similar, with small variations. Richness grows to larger values, resulting in higher complexity. The selection for mutualistic interactions is also stronger. All parameter values are the same as in the main results. (PNG) [file pcbi.1013402.s007.png]
