# Supplementary material for: Mutualism provides a basis for biodiversity in eco-evolutionary community assembly
Source: PLoS Comput Biol. 2025 Sep 2;21(9):e1013402. doi: 10.1371/journal.pcbi.1013402 (PMC12416848; doi:10.1371/journal.pcbi.1013402)
Supplement: Attachment — Submitted filename: Response_to_reviewers_final.pdf [file pcbi.1013402.s008.pdf]

Comments by the Editor:

Both reviewers felt the paper was interesting and important, and provided specific comments for the authors to revise. I have additional remarks for the authors: since the diverse interactions were important for network complexity and stability in your model, provided the details on the model parameter configurations, for example, those parameters like interaction parameters c_ij in the general LV model. How and why were some specific parameter values used in your simulations? Moreover, can you conduct extensive parameter space searching to check the robustness and consistency of your results and conclusions? I think these were important to reproduce, at least making the results become more convincing.

Response:

Dear Editor, Thank you for your consideration of our submission. We appreciate the interest and positive reviews on our manuscript.

We agree that conducting an extensive parameter space exploration would make our results and conclusions more robust. Following this suggestion by the editor, and similar suggestions by the reviewers, we have now conducted additional simulations varying the strength of interactions c_ij's (σ), the degree of inheritance (Δ), the initial biomass of the invader, as well as ecology and assembly processes happening at similar timescales. We have discussed these sensitivity analyses in the results and discussion sections and included several additional figures in our supplementary material illustrating the outcome of this parameter exploration. These and other changes suggested by the reviewers have helped us to improve our manuscript and have helped improve the robustness and consistency of our results.

To assess the sensitivity of our results to the interaction parameters c_ij, we performed further simulations with different values for the parameter σ in our model (the standard deviation of the distribution of c_ij's and which control their intensity). Our results are not qualitatively affected by these changes. We have added the following text to report the results from this analysis. [lines 232-235]:

"Our results are robust across a range of values of interaction strengths (c_ij's, p_ij's, and m_ij's). Simulations with σ values of 0.4, 0.6, and 0.8 for both Evo. and Inv. scenarios (n reps = 5 for each combination of σ and scenario type) yield qualitative similar results to those with σ =0.2 (S4 Fig)."

Results from the exploration of different values for other parameters are detailed below in the response to reviewers where they pointed out specific parameters that would be interesting to explore.

Below, we present a detailed point-by-point response to all the reviewers' comments (with our responses in blue text). Line numbers refer to the new clean version of the manuscript.

Comments by the reviewers:

Reviewer #1

The author considered generalized LV models of community assembly dynamics with mutualism, competition, predation, and evolution (speciation) and conducted numerical simulations. They found that highly mutualistic communities have complex and stable networks, causing a positive complexity-stability relationship. Because of the potential importance of eco-evolutionary dynamics, this kind of study is of interest for biologists. However, I feel the manuscript has some weaknesses.

Response: We appreciate the positive remarks by the reviewer and address the highlighted weaknesses below.

Major comments:

1. Here "evolution" is equivalent to "speciation", but other types of evolutionary dynamics can affect community assembly. For example, Kondoh (2003) Science showed adaptive foraging can produce a positive complexity-stability relationship. It would be great if the authors can add discussion on the potential difference between speciation and other evolutionary dynamics (adaptive trait changes that result in interaction changes, genetic drift, etc.).

Response: This is an interesting point. To emphasise the difference between speciation and other evolutionary dynamics we have extended our previous discussion on adaptive trait evolution, which was meant to go along these lines, with a deeper discussion of the potential effects of other forms of evolution. In particular, we have added the following text [lines 440-443]:

"Beyond the incorporation of trait adaptation mechanisms to complement our treatment of evolution as speciation events, other evolutionary mechanisms such as genetic drift and adaptive foraging can further promote the emergence of complexity in these model ecosystems [41]."

41. Kondoh M. Foraging adaptation and the relationship between food-web complexity and stability. Science. 2003;299(5611):1388–1391.

2. In the simulations, the authors assumed Delta = 5, but I was wondering how it affects the results. By changing the value of Delta, can the authors show the gradual change from "Evo." to "Inv."?

Response: We agree that exploring this gradual increase can provide further insight into the connections between Evo. and Inv. We have performed additional simulations showing that, by increasing Δ, *Evo.* results gradually become more similar to *Inv.* results. We present these results in the Supplementary Figure S5, and have added the following text to the manuscript to highlight them [lines 235-238]:

"Simulations with further degrees of inheritance strength (with $\Delta = \{15,25,35\}$ for the Evo. scenario, n=10 reps each), are consistent with the general trend from speciation to invasion, as evolutionary assembly results approach the ones of invasion assembly for lower inheritance degrees (S5 Fig)."

3. The difference between "Evo." and "Inv." is quite interesting. It would be great if the authors can add more discussion on the realistic scenario of "Evo." dynamics. Oceanic islands may produce community dynamics like "Evo." due to the scarcity of invasion events, but the timescale is a problem. Recent studies on eco-evolutionary dynamics consider rapid contemporary evolution, but speciation is usually excluded (e.g., Yamamichi et al. 2023 Ecol. Lett.). Thus, it will be interesting to check the timescale of speciation events in oceanic islands and the prevalence of mutualistic interactions in such isolated communities.

Response: It is very interesting to think of our results applied to oceanic island communities. We thank the reviewer for highlighting this connection. We found some previous work describing the presence of widespread mutualisms in oceanic island communities and the importance of these interactions for biodiversity. We also found some studies describing the importance of adaptive radiation in the community assembly of these islands. Using this information, we have added new text discussing these empirical patterns in light of our results.

We think this might be a promising venue for future research. The new text reads [lines 444-455]:

"Communities formed through a strong contribution of speciation events, often by the understudied process of adaptive radiation, are found on remote oceanic islands [42]. Across different islands, these communities are characterised by generalised mutualistic interactions and the presence of super-generalist species, and these interactions are fundamental to the maintenance of biodiversity and the weakening of latitudinal diversity gradients [43,44]. Despite their small area, these islands are responsible for a disproportionately high biodiversity globally [45]. These features are recapitulated in our results for communities assembled mainly by speciation events, which present a stronger selection for widespread mutualistic interactions, and these interactions in turn promote the emergence of more complex and diverse communities. Our results thus generate testable hypotheses for the mechanisms driving observed patterns of diversity in oceanic islands, a promising area of future research."

42. Cerca J, Cotoras DD, Bieker VC, De-Kayne R, Vargas P, Fernández-Mazuecos M, et al. Evolutionary genomics of oceanic island radiations. Trends Ecol. Evol. 2023;38(7):631–642.

43. Kaiser-Bunbury CN, Traveset A, Hansen DM. Conservation and restoration of plant–animal mutualisms on oceanic islands. Perspect. Pl. Ecol. Evol. Syst. 2010;12(2):131–143.

44. Delavaux CS, Crowther TW, Bever JD, Weigelt P, Gora EM. Mutualisms weaken the latitudinal diversity gradient among oceanic islands. Nature. 2024;627(8003):335–339.

45. Cowie RH, Holland BS. Dispersal is fundamental to biogeography and the evolution of biodiversity on oceanic islands. J. Biogeogr. 2006;33(2):193–198.

Further, in response to a similar comment made by the other reviewer regarding the separation of ecological and evolutionary timescales (see response below), we performed further simulations exploring different extents of separation between timescales to dig deeper into the effects of this separation for community assembly in our model. We included new simulation results without separation of timescales in (Suplemmentary Figure S6) [lines 472-473]:

"Additional simulations with several rates of out-of-equilibrium assembly events revealed similar results to those observed in our main simulations (S6 Fig)"

Minor comments:

Figure 1 legend: Does "richness" in (A) represent the number of species? It would be better to write the definition of "connectance" in the legend as well.

Response: Apologies for the lack of clarity. We added explanations for them: [lines 123-126]

"Across assembly time (1000 assembly events), both evolved and invaded communities showed a steady increase in species richness (number of species) and a slow decrease in connectance (fraction of realised interactions from all possible ones), which resulted in an overall increase in complexity."

Figure 2A: It would be better to move the legend down a bit to avoid the overlap with the red lines.

Response: We have updated the figure with the legend in a new position.

Figure 2 legend: It will be nice to clarify that the green and yellow lines are overlapping in Fig. 2A.

Response: We have added this clarification.

Figure 3: I was wondering whether the authors really need colors here or not.

Response: The colours are not necessary to understand the overall distribution of the eigenvalues. However, we opted to leave the colours in as they provide additional information on the mixing across samples.

L431: have a differ from the parent a number → have a difference from the parent in a number

Response: Corrected.

Reviewer #2

The authors introduce an eco-evolutionary assembly model, where ecological communities emerge either via speciation or immigration events (or a mix of both). The key novelty of the study is the mix of interaction types, meaning that assemblages consist of antagonistic, competitive and mutualistic interactions, whereas previous studies typically focused on single interaction types in isolation (such as food webs or pollination networks). The authors demonstrate that evolution promotes higher proportions of mutualism and that mutualistic interactions in turn enhance the dynamical stability of assembled communities. Overall, I truly enjoyed reading this manuscript. It is very well written, the story follows a clear logic and the results are highly interesting. I think it is a good fit for PLOS Computational Biology. My comments are mostly minor and listed below in chronological order.

Response: We thank the reviewer for their positive comments on our manuscript and are happy they have enjoyed reading it. We appreciate the detailed review provided and address their suggestions below.

line 17-20: I don't understand this statement. Yes, pairwise mutualistic interaction create positive feedback, but the same is true for pairwise competitive interactions. So why should a higher proportion of competitive interactions favour stability?

Response: We have amended this sentence to improve its clarity [lines 17-21]:

"However, these results are mixed in the literature, with other studies showing that strong positive feedbacks from mutualistic interactions may destabilise communities comprised of multiple interaction types, thus making a higher proportion of competition or antagonism favourable to stability".

Just to provide some explanation, in our model competitive interactions do not create a positive feedback in the sense we refer to it in our manuscript. Suppose there are two species engaging in a (Type I) mutualistic interaction. They continually benefit each other via the positive interaction, resulting in higher abundances and an even stronger interaction, which constitutes the positive feedback at an individual level. In competitive interactions on the other hand, when two species interact, they promote their deaths, which is a cost by itself and results in lower abundances and weaker overall interaction. The strong positive feedback comes from repeatedly interacting without any cost and increasing the intensity of interaction in the mutualistic case. Type II functional responses on the other hand implement the cost of,

individually, waiting for a period between each interaction (the parameter h, generally called handling time), using delays to prevent the positive feedback.

- line 44-46: Maybe split the list of references in this sentence, so it becomes clear which ones refer to mutualistic systems and which ones refer to food webs. Also, consider adding the following references:

Becker, L., Blüthgen, N., & Drossel, B. (2022). Stochasticity leads to coexistence of generalists and specialists in assembling mutualistic communities. The American Naturalist, 200(3), 303-315.

Metz, T., Blüthgen, N., & Drossel, B. (2023). Shifts from non-obligate generalists to obligate specialists in simulations of mutualistic network assembly. Oikos, 2023(7), e09697.

Allhoff, K. T., Ritterskamp, D., Rall, B. C., Drossel, B., & Guill, C. (2015). Evolutionary food web model based on body masses gives realistic networks with permanent species turnover. Scientific reports, 5(1), 10955.

Response: We separated the references and added these to the list. Thank you for the interesting suggestions, they are in line with the direction we are pointing out.

- lines 48-55, as well as lines 75-77: This reminded me of the study by Morris et al. (2021), which also focusses on network assembly via a mix of mutations/speciations and invasions. Maybe it's worth referencing?

Morris, J. R., Allhoff, K. T., & Valdovinos, F. S. (2021). Strange invaders increase disturbance and promote generalists in an evolving food web. Scientific Reports, 11(1), 21274.

Response: That is a good reference for considering assembly by either evolution or invasion, and we were not aware of it. We included the reference in both sentences. Thank you.

- line 85-87, as well as lines 181-184: I was very surprised by the finding that mutualism acts as a stabilising force. As mentioned above, mutualism creates positive (=self-reinforcing) feedback, so how can it be stabilising? As far as I know, systems actually tend to become LESS stable when mutualism outweighs antagonism (see for example Yacine and Loeuille 2022). Is this due to the functional response? Please clarify.

Yacine, Y., & Loeuille, N. (2022). Stable coexistence in plant-pollinator-herbivore communities requires balanced mutualistic vs antagonistic interactions. Ecological Modelling, 465, 109857.

Response: Yes, as the reviewer anticipates this is due to the functional response used in our study (Type II), which avoids the strong positive feedback created by mutualistic interactions when modelled using Type I responses. The latter is not only destabilising but also unrealistic as it implies indefinite resource consumption. At the individual level, the interpretation of Type II functional response is that each interaction promotes a 'relaxation' period during which the individual does not interact (usually defined as a handling time in consumer-resource interactions). This change prevents large abundance fluctuations and infinity.

We updated the text to make this point clearer: [lines 86-67]

"Additionally, mutualism with Type II functional response enhances stability against perturbations by shifting the trajectories of abundances to more stable regions."

And [lines 246-249]:

“The presence of a large proportion of mutualism, both under ecological and evolutionary assembly, considerably shifts the distributions to the negative, 'more stable' zone of the eigenvalue space (Fig 3). A Type II functional response might be relevant in producing this result.”

- Along similar lines, Maliet et al (2020) showed that antagonistic interactions tend to foster species and trait diversity, while mutualistic interactions tend to reduce diversity, which seems to be in contrast to the key results presented here. Why these contrasting results? I think this apparent contradiction deserves to be discussed.

Maliet, O., Loeuille, N., & Morlon, H. (2020). An individual-based model for the eco-evolutionary emergence of bipartite interaction networks. Ecology Letters, 23(11), 1623-1634.

Please clarify.

Response: We do not think this is an actual contradiction but contrasting results emerging from contrasting modelling approaches. We added a discussion about this issue, using Maliet et al. (2020) as an example [lines 494-506]:

“The stabilisation of mutualistic interactions through the use of Type II functional responses points to an important reason for the apparent contradiction between our results and those from previous studies, which vary in assumptions and modelling frameworks used. For example, Coyte et al. (2015) [12] concludes that mutualisms are destabilising, but their model uses a Type I functional response that generates positive feedbacks between mutualistic species. Maliet et al. (2020) [52] conclude that mutualisms reduce diversity, but they use an individual-based model with fixed population size and interactions between explicitly defined individuals. We argue that a way forward to address apparent contradictions between modelling approaches is to map the different conditions and assumptions made by different models into a bigger picture of distinct scenarios with potentially different results. This will provide clarity regarding the conditions under which different interaction types can stabilise or destabilise system dynamics.”

12. Coyte KZ, Schluter J, Foster KR. The ecology of the microbiome: networks, competition, and stability. Science. 2015;350(6261):663–666.

52. Maliet O, Loeuille N, Morlon H. An individual-based model for the eco-evolutionary emergence of bipartite interaction networks. Ecol. Lett. 2020;23(11):1623–1634.

- Fig 1: Why is there no "evo high M" scenario? Could that be implemented as well?

Response: This scenario was not implemented because it would involve imposing external constraints on the proportion of mutualisms. Part of how we define the evolution scenario is letting the proportions evolve naturally. Also, evolution already produces a very high proportion of mutualisms, so it wasn’t necessary to have an additional scenario enforcing this.

- Fig 1 and 2: Consider refering to line colous in the text. Also, please check whether your choice of colours works for colour-blind people.

Response: We thank the reviewer for this suggestion. We updated our figures with a colour-blind friendly palette. Additionally, we now refer to the colours in the results section where we explained each scenario.

- The error bars in most of the figures are very small, indicating that a given parameter combination would always lead to more or less the same network structure. Is this correct? If yes, is it possible to visualise these networks? How do they look like?

Response: There is considerable variability across simulations in terms of the size and structure of the networks, especially in the evolution scenarios. We initially thought of presenting this variability in the plots as the standard deviations represented by the vertical bars, however, some of them were very large and made the figures hard to understand. In the interest of clarity, we opted to present the variability using the measure of standard error instead, which is the standard deviation divided by the square root of the number of samples. We believe this measure shows how accurate is our estimation of averages (and they are small). The range of variability across network samples can be observed by looking at Fig 6 in the main text, which shows richness and connectance pairs for each individual sample at the end of simulations. The pure evolution case (blue) features values of richness between around 500 and 700, and connectance between around 0.05 and 0.35. This is the same scenario as the *Evo.* in Fig 1 (blue). In Fig 1A and Fig 1B we can see, in the last step, their corresponding averages with very small errors (for 20 samples).

In the methods section, we added an explanation of the error bars for further clarification [lines 690-691]:

"The standard error indicates the uncertainty in our estimations of averages from our sets of samples."

- caption of Fig 6: I think there is a typo: Mixed 0.2 should be 20% invasion, right?

Response: Corrected.

- The discussion provides an excellent overview of the key results and their implications. However, I sometimes miss some information of how these results emerge from the underlying model assumptions. In general, I am not a big fan of putting the methods at the end (but I guess this is a Journal requirement?). I found it difficult to understand the results while reading the manuscript for a first time, and had to read it a second time, now going back and forth between methods and results section, before it finally made sense. A bit more info about the methods earlier in the manuscript might help.

Response: We are sorry to hear it was hard to understand the manuscript without going back and forth to the methods. We have added to the results section a sentence including two important model assumptions that were previously absent, and hope this improves the readability of the paper: [lines 114-117]

"We adopted Type II functional responses that enable saturation of positive interactions (consumers and mutualists) and analyse a regime of high average interaction weights (see Methods for details)."

- line 305: another typo: "Swueis" should be "Suweis"

Response: Corrected.

- line 483: I'm not sure I understand why you made pij+=pij- if pij+>pij-. Doesn't this translate into the absence of convergence efficiency losses, so that every bit of resource biomass that is consumed turns into predator biomass?

Response: We assume resource biomass is turned into consumer biomass with an efficiency that is incorporated into the pij coefficients. However, due to the random draw of pij coefficients, there could be instances in which the biomass conversion efficiency is larger than 1 (i.e. pij+>pij-) thus creating an energetic imbalance. We correct this by imposing the constraint referred to by the reviewer. To make this point clearer in the text, we have included the following explanation: [lines 681-682]:

"This condition is introduced to prevent the biomass conversion efficiency for consumers from exceeding 1."

- lines 400-408: I am not sure I understand this correctly. So a given species that is perfectly viable with a given set of interactions can be pushed to towards extinction by adding more positive interactions? Why? Why should the species suddenly suffer when it gets even more resources? From a technical perspective, I see that this assumption is probably needed to avoid ever growing networks, but from an ecological perspective I think it is problematic and needs additional clarification.

Response: We do not think this assumption is needed to avoid ever growing networks. We just assume a trade-off between establishing additional interactions and intrinsic growth. The rationale for this is that developing traits to engage in new interactions is costly and this cost is paid at the expense of other reproductive activities. It can either decrease the effect of independent sources of growth, increase mortality rates, or even demand more energy/resources for maintenance. Thus, the outcome of this is not to push the species towards extinction but making it more dependent on interactions. *'Why should the species suddenly suffer when it gets even more resources?':* By getting more resources, the species is being benefitted, but it also 'suffers' a cost to maintain its ability of growing when interaction partners are absent.

- line 414: you assume that the system reaches a steady state before the next species is added, which basically reflects a seperation of ecological versus evolutionary timescales. What is the reasoning behind this assumption? Is it necessary? How would the results change when overlapping timesclapes are taken into account? I'm asking because there is ample evidence for rapid evolution nowadays, see for example:

Hairston Jr, N. G., Ellner, S. P., Geber, M. A., Yoshida, T., & Fox, J. A. (2005). Rapid evolution and the convergence of ecological and evolutionary time. Ecology letters, 8(10), 1114-1127.

Thompson, J. N. (1998). Rapid evolution as an ecological process. Trends in ecology & evolution, 13(8), 329-332.

Response: This is an excellent point made by the reviewer. To address this, we have explored additional simulations with rapid evolution, by allowing assembly events in varied frequencies instead of waiting until ecological equilibrium (newly added Supplementary Figure S6). The results remain mainly the same, however a very high frequency of speciation events led to a dramatic decline of connectance and thus complexity. We added a paragraph in the discussion commenting on this result and on rapid evolution, also citing the suggested papers: [lines 468-478]

"Another mechanism with the potential to impact community structure is the rapid evolution of ecological interactions, in which evolutionary changes happen at ecological timescales [46,47]. Therefore, the addition of new species during the ecological transient dynamics, before equilibrium, might result in different community assembly patterns. Additional simulations with several rates of out-of-equilibrium assembly events revealed similar results to those observed

in our main simulations (S6 Fig). However, speciation events occurring at high frequency resulted in a significant loss of interactions and a consequent drop in the values of community complexity. These results suggest that rapid evolution might strongly impact ecological interactions and the complexity of ecological systems. Given the empirical relevance of rapid evolutionary changes, this phenomenon deserves further investigation in future studies."

46. Thompson JN. Rapid evolution as an ecological process. Trends Ecol. Evol. 1998;13(8):329–332.

47. Hairston Jr NG, Ellner SP, Geber MA, Yoshida T, Fox JA. Rapid evolution and the convergence of ecological and evolutionary time. Ecol. Lett. 2005;8(10):1114–1127.

- line 422: Shouldn't the mutation rate somehow depend on biomass densities? I guess that larger populations have a higher probability to generate mutants, right?

Response: This is an interesting point raised by the reviewer. To assess the effects of biomass-dependent mutation rates we ran further simulations of evolutionary assembly with speciation rates as a function of species densities (Supplementary Figure S7). The results remain very similar and are qualitatively the same. We added the following text to report this finding [lines 239-240]:

"Additionally, assuming speciation rates to be proportional to abundance in assembly by evolution, instead of uniform, did not qualitatively altered our results (S7 Fig)."

- line 430-432: Check grammar in this sentence.

Response: Corrected.

- line 497: As far as I know network modularity is usually calculated via simulated annealing. Is this also done in the python package that you use here? If yes, maybe explain a bit how that works. If not – why not using simulated annealing?

Response: We are aware that simulated annealing is a method commonly used to calculate modularity. In this study we opted for a comparably efficient method called the Louvain algorithm which is grounded on greedy optimisation instead (doi:10.1088/1742-5468/2008/10/P10008). We added this information to the text and cited the method: [lines 702-705]

"We manipulated networks using the python package NetworkX [59]. Optimal community partitions for modularity calculation were obtained using the Louvain greedy optimisation algorithm [60]."

59. Hagberg A, Swart PJ, Schult DA. Exploring network structure, dynamics, and function using NetworkX. Proc 7th Python Sci Conf. 2008;p. 11–15.

60. Blondel VD, Guillaume JL, Lambiotte R, Lefebvre E. Fast unfolding of communities in large networks. J. Stat. Mech.: Theory Exp. 2008;2008(10):P10008.

- line 506: typo "Erdos-Renyi" should be "Erdős–Rényi". Also, a reference is missing here.

Response: Corrected.
