# Supplementary material for: Mutualism provides a basis for biodiversity in eco-evolutionary community assembly
Source: PLoS Comput Biol. 2025 Sep 2;21(9):e1013402. doi: 10.1371/journal.pcbi.1013402 (PMC12416848; doi:10.1371/journal.pcbi.1013402)
Supplement: Attachment — Submitted filename: Response_to_reviewers_2nd_revision.pdf [file pcbi.1013402.s009.pdf]

Additional Editor Comments:

Dear Author: Thank you for your careful revision and both reviewers now had made additional but minor comments for you to improve. Please revise the paper throughout again before final acceptance.

Response: Dear editor, thank you once again for managing the review of our manuscript. We have now revised the latest version of our paper addressing the additional comments raised by the reviewers during this second round of reviews. We appreciate the important clarifications raised by reviewers and updated the text with the required changes, as detailed below in our point-by-point response.

Reviewers' comments:

Reviewer #1: The manuscript was improved through revision, but I still have some comments.

Response: We thank the reviewer for the time and effort devoted to this additional review and the important clarifications indicated by their comments.

Abstract: It will be great if the authors can clarify that "an evolutionary process" is speciation and "a purely ecological assembly" is via migration here. Then readers will be able to understand what "in isolation" means easily.

Response: Thank for raising these clarifications. We fully agree that these improve the clarity of the paper. We are now more specific about the ecological and evolutionary processes in the abstract based on this suggestion. It now reads:

"We show that evolution by speciation enhances the emergence of such conditions compared to a purely ecological assembly scenario of repeated invasions by migrating species."

Author summary: It may be better to avoid using "predator-prey" and "consumer-resource" as some readers may wonder the difference between them. What about removing "consumer-resource" and using "predator-prey" throughout the author summary?

Response: We thank the reviewer for pointing this out. To maintain consistency throughout the manuscript, we opted for the term 'consumer-resource' instead of 'predator-prey' as the latter was only used in this instance. This sentence of the author summary now reads:

"Most research into the mechanisms of biodiversity considers different interaction types (mutualism, competition, consumer-resource) in isolation in either ecological or evolutionary contexts."

L12: The term "eco-evolutionary" is sometimes very confusing due to the time-scales of dynamics as pointed out by Bassar et al. (2021) Towards a more precise – and accurate – view of eco-evolution. Ecology Letters. It will be helpful to clarify the term here.

Response: We thank the reviewer for highlighting this. To avoid confusion throughout the manuscript, we now clarify that when we use the term "eco-evolutionary" when speaking of assembly processes, we are referring to an assembly process that is influenced by both ecological and evolutionary mechanisms, even if they happen at different timescales, which is the scenario analysed in our main results. However, in the supplementary material (Fig S6), we checked that our analysis is preserved when ecological and evolutionary processes happen in similar timescales. The updated version of this statement now reads:

"This raises the question of how different complex structures of species interaction networks emerge through eco-evolutionary processes (i.e. driven by both ecological and evolutionary mechanisms, even if they happen at different timescales) while maintaining species persistence and ecosystem stability." (L10-14).

L18-19, 32: Readers may wonder linear functional responses destabilize dynamics with mutualism whereas saturating functional responses stabilize dynamics. It would be better to clarify the relationship between functional responses and stability.

Response: We agree that this clarification would make it clearer for the reader to understand the differences between functional responses early in the text. We have added sentences further explaining how the functional response can stabilise the dynamics of population models:

"These contrasting results partly stem from the use of different functional responses defining interactions across modelling studies. A linear Type I response tends to destabilise mutualistic dynamics by generating positive feedback loops that can lead to unbounded population growth. In contrast, nonlinear saturating responses (e.g., Type II) can mitigate these effects by introducing consumption limits, thereby dampening positive feedback and promoting stability." [L22-28]

L32, 88: It might be helpful to clarify that "saturating" and "Type II functional responses" are equivalent.

Response: We have added "(e.g. Type II)" to L32 (now L37), and "Type II saturating functional response" to L88 (now L95) to make this equivalence clearer.

L33: It will be nice to explain the term "priority effects" for readers who are not familiar with it.

Response: We agree with this suggestion. We have re-phrased this, adding a brief definition of priority effects. In L39-42:

"In addition, priority effects, in which the order of species arrival influences the outcome of assembly, constitute a major driver of community assembly. Random differences in colonisation history are known to cause large variations in community composition [19], which promote critical selection pressures for eco-evolutionary mechanisms to act on [20]."

L147: richness (A) -> richness (S) (A)

Response: Added.

L148: connectance (B) -> connectance (C) (B)

Response: Added.

L150: S and C should be in Italic.

Response: Corrected.

L235: What does "n reps" mean?

Response: It means just the number of repetitions of simulations executed for each scenario. We changed '*n reps = 5*' to '*5 samples*' so the meaning is clearer.

L443-444: Did Kondoh (2003) consider genetic drift? How does drift promote the emergence of complexity in communities?

Response: Sorry, this was a mistake. We removed the mention to genetic drift and maintained only adaptive foraging, which is the subject of Kondoh's paper. Thanks for pointing it out.

Reviewer #2: Once again, I very much enjoyed reading this very interesting manuscript. The previous version was already of very high quality but the manuscript nevertheless improved during peer review.

Response: We thank the reviewer for the kind words and the favourable assessment of our work. We are also grateful for the review and sharing the ideas below about feedbacks in competitive interactions. We found the discussion topic fascinating and provide our contribution to it below. It would have been nice to held discussions in person!

I have only one small remark on competitive feedback loops. The authors write in their response that "competitive interactions do not create a positive feedback". I disagree. Competitive interactions (in isolation) always result in positive (=self-reinforcing) feedback, simply because the product of two negative numbers is a positive number. For clarification, imagine two competing populations at equilibrium. If species 1 experiences a decline in abundance due to an external disturbance, then this decline will lead to a reduction in competitive pressure on species 2, so species 2 can reach higher abundances, which will in turn translate into an increased competitive pressure on species 1, thus reinforcing the initial disturbance. In the absence of all other forces, this feedback loop is thus clearly destabilising, because it pushes species 1 towards extinction and species 2 towards high abundances.

However, other forces, such as selfregulation via intraspecific competition and/or interactions with other species, will most likely prevent too high (and hence unrealistic) abundances of species 2. In general, I would argue that it is not very insightful to look at single feedback loops

in isolation, given that feedback loops usually act in concert and potentially balance each other. For more context, I recommend to read the following paper: Levins, R. (1974). Discussion paper: the qualitative analysis of partially specified systems. Annals of the New York Academy of Sciences, 231(1), 123-138.

Plase note that I bring this up in response to the author's response, but I don't think this requires additional revision of the text. In fact, all my comments have been thoroughly adressed. I do not have any further suggestions for improvement and recommend acceptence of the article as is. I thank the authors for their extensive and insightful replies!

Response: We fully agree with the reviewer's analysis and found the example presented by the reviewer engaging and relevant. In our previous explanation, we tried to convey a perspective of individual-level positive reinforcement that is unstoppable and necessarily destabilises the system. In the case of mutualistic interactions with Type I functional responses, the underlying assumption is that the same individual of a given population can interact again and again without wait with many different individuals of the other (interacting) population, producing more individuals in the process that in turn interact again and again, without costs or delays. However, in competition, individuals interact and are removed from the population (i.e. they die), thus preventing local amplification of the interaction and not necessarily causing a disruption. In a dynamical systems perspective, on the other hand, we completely agree with the reviewer and with their description of how to correctly define positive feedbacks. We wish to thank the reviewer again for taking the time to read our comments and share this clarification.
